# Anti-Inflammatory Activity of Compounds Isolated from *Digitalis purpurea* L. in TNF-α/IFN-γ-Induced HaCaT Keratinocytes and a Three-Dimensionally Reconstructed Human Skin Model

**DOI:** 10.3390/ijms26167747

**Published:** 2025-08-11

**Authors:** Linsha Dong, Hwan Lee, Zhiming Liu, Eun-Rhan Woo, Dong-Sung Lee

**Affiliations:** 1 College of Nursing, Qingdao Binhai University, Qingdao 266555, China; donglinsha011@outlook.com; 2 Research Institute of Pharmaceutical Sciences (RIPS), College of Pharmacy, Chosun University, Dong-gu, Gwangju 61452, Republic of Korea; ghksdldi123@hanmail.net (H.L.); liuzhiming@ytu.edu.cn (Z.L.); wooer@chosun.ac.kr (E.-R.W.); 3 Key Laboratory of Molecular Pharmacology and Drug Evaluation, Ministry of Education, School of Pharmacy, Yantai University, Yantai 264005, China

**Keywords:** desrhamnosyl acteoside, *Digitalis purpurea*, keratinocyte, atopic dermatitis, NF-κB, JAK/STAT, 3D human skin model

## Abstract

Atopic dermatitis (AD) is a chronic, relapsing inflammatory skin disorder affecting 10–20% of the population. In this study, we investigate the anti-inflammatory effect on the skin of eight compounds isolated from *Digitalis purpurea* L., using tumor necrosis factor-α (TNF-α)/interferon-γ (IFN-γ)-stimulated human keratinocytes (HaCaT cells) and a three-dimensional (3D) reconstructed human skin model. Among the tested compounds, desrhamnosyl acteoside exhibited the most potent activity, significantly reducing the secretion of pro-inflammatory cytokines (IL-6, IL-8) and chemokines (CCL17, CCL22), suppressing the expression of inflammatory proteins, and modulating key signaling pathways, including NF-κB, JAK2/STAT1, and MAPK. Notably, this is the first report demonstrating that desrhamnosyl acteoside simultaneously targets all three pathways, indicating a multi-modal mechanism distinct from conventional single-target approaches. In the 3D skin model, desrhamnosyl acteoside further exhibited barrier-protective effects by downregulating inflammatory mediators and upregulating epidermal differentiation markers such as involucrin and loricrin. These findings reveal a previously uncharacterized phytochemical with dual anti-inflammatory and barrier-restorative activities, supporting its potential as a novel therapeutic candidate for AD and other inflammatory skin diseases.

## 1. Introduction

Atopic dermatitis (AD) is a chronic inflammatory skin disorder characterized by pruritic eczematous lesions and epidermal barrier dysfunction. It arises from a multifactorial interplay of genetic predisposition, immune dysregulation, and environmental triggers [1,2]. Although conventional treatments such as emollients, topical corticosteroids, and systemic immunosuppressants can provide symptomatic relief, their long-term use is often limited due to adverse effects (e.g., skin atrophy and rebound flares) and incomplete disease control [3]. These limitations have stimulated growing interest in natural compounds with multi-targeted anti-inflammatory actions and favorable safety profiles, such as quercetin, resveratrol, and curcumin [4,5]. While various inflammatory skin diseases share overlapping mechanisms, AD remains one of the most prevalent and clinically burdensome conditions, particularly due to its chronic relapsing nature and strong association with skin barrier disruption. We therefore selected AD as the primary disease model in this study to allow for a focused investigation with translational relevance. Other inflammatory dermatoses, such as psoriasis and contact dermatitis, involve distinct immune axes—including Th17/Th1-mediated pathways and NLRP3 inflammasome activation. Although recent advances in biologics (e.g., IL-17/IL-23 inhibitors) and small-molecule drugs (e.g., JAK-STAT inhibitors) have transformed treatment strategies for moderate-to-severe inflammatory skin disorders, therapeutic resistance, inter-patient variability, and long-term safety concerns remain major challenges [6,7]. Emerging therapeutic approaches include nanocarrier-mediated delivery systems, multi-omics-based precision strategies, and microbiome-targeted interventions, each offering new possibilities for personalized dermatologic care [8,9].

The epidermal barrier comprises multiple components, including structural proteins such as filaggrin and involucrin, tight junction proteins, and innate immune effectors [10]. These elements cross-link to form a scaffold within the extracellular lipid matrix, maintaining skin hydration, pH, and structural integrity [11]. Barrier disruption, a hallmark of AD, increases skin permeability, facilitates the penetration of environmental allergens, and promotes inappropriate immune activation. This, in turn, leads to both localized inflammation and systemic immune responses. Therefore, restoring barrier function through modulation of skin structural proteins represents a promising therapeutic strategy for AD [12]. Keratinocytes, the predominant cells of the epidermis, play a pivotal role in skin barrier formation and immune surveillance. They express pattern recognition receptors that detect microbial components and environmental stimuli, initiating innate immune responses [13,14]. Upon activation, keratinocytes secrete various pro-inflammatory mediators, including cytokines, chemokines, antimicrobial peptides, growth factors, and lipid-derived molecules, which collectively orchestrate immune cell recruitment and amplification of skin inflammation [15,16].

In inflammatory skin disorders, pro-inflammatory cytokines, chemokines, and their upstream signaling pathways interact in a tightly regulated cascade. Cytokines such as TNF-α and IFN-γ are released early in response to environmental triggers, enhancing the expression of adhesion molecules and facilitating leukocyte infiltration [17,18]. Chemokines then guide immune cells—including neutrophils, monocytes, and T lymphocytes—to inflamed tissue sites. These processes are regulated by key intracellular signaling pathways, particularly NF-κB, JAK/STAT, and MAPK, which control the transcriptional activation of inflammatory genes. Dysregulation of these pathways contributes to chronic inflammation and disease progression in various dermatoses [19].

*Digitalis purpurea* L., a flowering plant native to Europe, is widely cultivated as a medicinal resource. It is known for its ability to biosynthesize cardiac glycosides such as digitoxin and digoxin, which are clinically used for managing systolic heart failure and controlling ventricular rate in atrial fibrillation. Due to their structural complexity, these compounds are difficult to synthesize chemically and are still primarily obtained through plant extraction [20,21]. In our previous work, we isolated and structurally characterized several compounds from *D. purpurea*, some of which displayed promising anti-inflammatory activity. For example, purpuride A was shown to inhibit inflammatory responses in macrophage models [22]. However, the protective effects of other compounds isolated from *D. purpurea* against skin inflammation remain largely unexplored. In the present study, we aim to evaluate the anti-inflammatory and skin barrier–restorative effects of eight compounds isolated from *D. purpurea*, using TNF-α/IFN-γ-stimulated human epidermal keratinocytes and a three-dimensional (3D) reconstructed human skin model. Our goal was to identify potential therapeutic candidates for AD by elucidating the molecular mechanisms involved in keratinocyte inflammation and epidermal barrier disruption.

## 2. Results

### 2.1. Identification of Eight Compounds Isolated from D. purpurea L.

**Figure 1:**
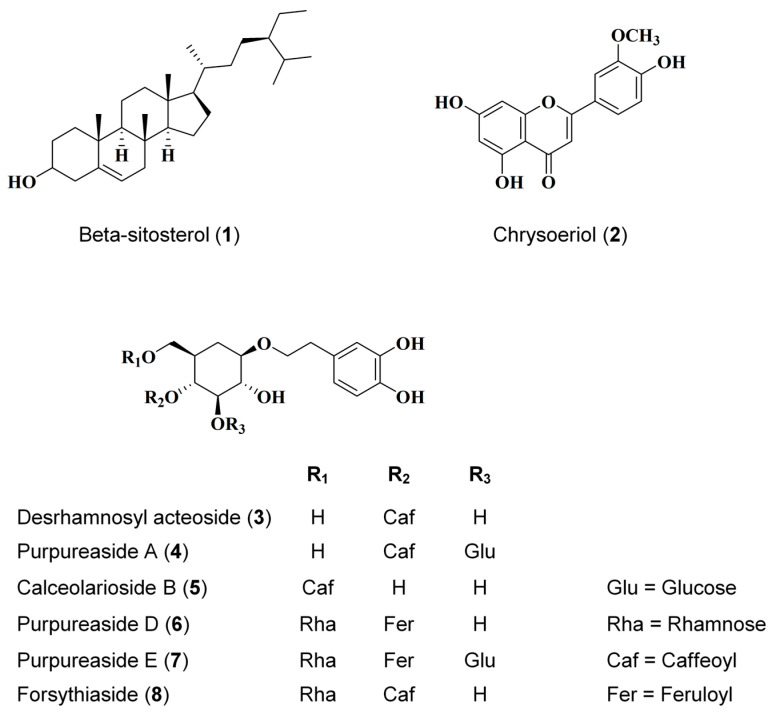
Structure of eight isolated compounds from *Digitalis purpurea* L.

After separation and identification, the following eight compounds were obtained from *D. purpurea* L.: beta-sitosterol (**1**), chrysoeriol (**2**), desrhamnosyl acteoside (**3**), purpureaside A (**4**), calceolarioside B (**5**), purpureaside D (**6**), purpureaside E (**7**), and forsythiaside (**8**). Their chemical structures are shown in Figure 1.

### 2.2. Cytotoxicity of Eight Compounds Isolated from D. purpurea L. in HaCaTs

**Figure 2:**
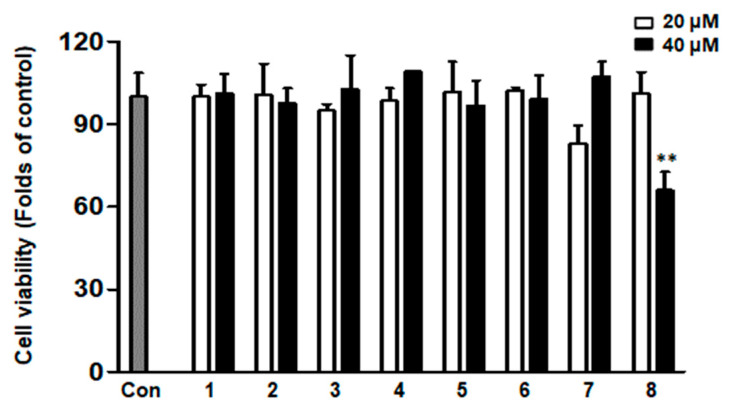
Cytotoxicity of isolated compounds from *Digitalis purpurea* L. in HaCaTs. The cells were pre-treated with compounds **1**–**8**, 20 μM and 40 μM for 24 h. Cytotoxicity were tested by MTT assay. ** *p* < 0.01, vs. the Con group (untreated control, gray column). Data are presented as mean ± SD (*n* = 3 independent experiments).

First, we examined the cytotoxicity of eight compounds isolated from *D. purpurea* L. on HaCaTs. The MTT results shown in Figure 2 indicate that 20 and 40 μM are safe concentrations for HaCaTs for compounds **1**–**7**, whereas 10 and 20 μM are safe for compound **8**.

### 2.3. Effects of Eight Compounds Isolated from D. purpurea L. on IL-6/IL-8 in TNF-α/IFN-γ HaCaTs

**Figure 3:**
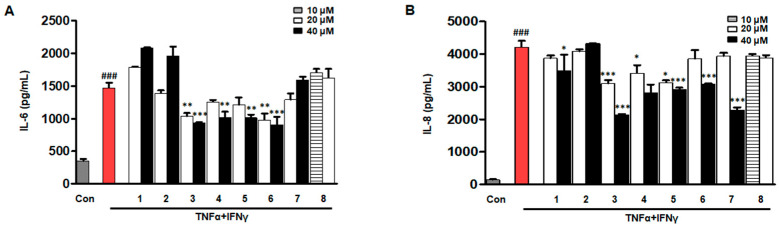
Initial screening of the effects of compounds isolated from *Digitalis purpurea* L. on IL-6 (**A**) and IL-8 (**B**) production in TNF-α/IFN-γ-stimulated HaCaT keratinocytes. Cytokine levels were quantified from culture supernatants. Cells were pretreated with each compound for 3 h and subsequently stimulated with TNF-α/IFN-γ for 24 h. Data are presented as mean ± SD (*n* = 3 independent experiments). ^###^ *p* < 0.001 vs. Con group (gray column). * *p* < 0.05, ** *p* < 0.01, *** *p* < 0.001 vs. TNF-α/IFN-γ-treated group (red column). “Con” indicates the untreated control group.

Interleukin 6 (IL-6) and Interleukin 8 (IL-8) are multifunctional cytokines involved in inflammation, the acute-phase response, hematopoiesis, and immune regulation [23,24]. HaCaT cells were stimulated with TNF-α/IFN-γ for 24 h. As shown in Figure 3, compounds **3**, **4**, **5**, and **6** significantly inhibited the secretion of both IL-6 and IL-8 compared to the TNF-α/IFN-γ-treated group. Compound **1** and compound **7** showed a statistically significant reduction in IL-8 levels at 40 µM, but neither exhibited a consistent or dose-dependent effect, and they did not reduce IL-6 secretion. Compounds **2** and **8** had no significant effect on either cytokine. Therefore, compounds **3**, **4**, **5**, and **6** were selected for subsequent experiments due to their consistent and robust inhibitory activities on both IL-6 and IL-8.

### 2.4. Effects of Compounds ***3***–***6*** on RANTES and MDC in TNF-α/IFN-γ-Induced HaCaTs

**Figure 4:**
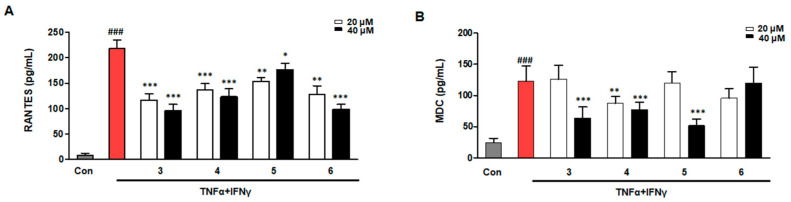
Inhibitory effects of compounds **3**–**6** on RANTES and MDC secretion. The RANTES (**A**) and MDC (**B**) levels were measured using the cell culture supernatants. The cells were pre-treated with compound **3** for 3 h and then stimulated with TNF-α/IFN-γ for 24 h. Data are represented as the mean ± SD (*n* = 3 independent experiments). ^###^ *p* < 0.001 vs. Con group (gray column). * *p* < 0.05, ** *p* < 0.01, *** *p* < 0.001 vs. TNF-α/IFN-γ-treated group (red column). “Con” indicates the untreated control group.

Keratinocytes induce the thymus to release activated regulatory chemokine (TARC/CCL17) and macrophage-derived chemokine (MDC/CCL22), which are crucial for the infiltration of inflamed tissues by Th2 cells [25]. The TNF-α/IFN-γ group was set up by treating keratinocytes with TNF-α/IFN-γ. The effects of compounds **3**, **4**, **5**, and **6** on the secretion of regulated on activation in normal t-cell expressed and secreted (RANTES) and MDC, when compared with the TNF-α/IFN-γ group, are shown in Figure 4. All four compounds inhibited RANTES production as compared to the TNF-α/IFN-γ group. Compound **6** had no significant effect on MDC levels when contrasted with the TNF-α/IFN-γ group.

### 2.5. Effects of Compounds ***3***–***6*** on ICAM-1 Expression and NF-κB (p65) Binding Activity in TNF-α/IFN-γ HaCaTs

**Figure 5:**
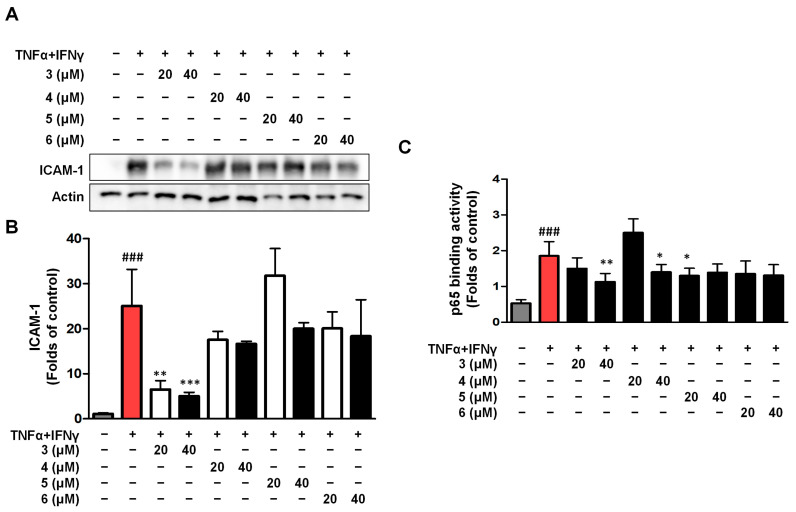
Compounds **3**–**6** suppress TNF-α/IFN-γ-induced ICAM-1 expression (**A**,**B**) and NF-κB Activation (**C**). The cells were pre-treated with compound **3** for 3 h and then stimulated with TNF-α/IFN-γ for 24 h. The expression of ICAM-1 was measured using Western blot. The p65 binding activity was detected by the p65 ELISA kit. The data are represented as the mean ± SD (*n* = 3 independent experiments). ^###^ *p* < 0.001 vs. untreated control group (gray column). * *p* < 0.05, ** *p* < 0.01, *** *p* < 0.001 vs. TNF-α/IFN-γ-treated group (red column).

Intercellular Cell Adhesion Molecule-1 (ICAM-1) is a cell surface glycoprotein and adhesion receptor that regulates the recruitment of white blood cells from circulation to inflammatory sites [26,27]. The effects of compounds **3**, **4**, **5**, and **6** on ICAM-1 expression were detected using Western blot (Figure 5A,B). The results of ICAM-1 showed that only compound **3** inhibited ICAM-1. We also tested the binding activity of p65 to its corresponding DNA in the cell nucleus for compounds **3**–**6**, and the results showed that compound **3** had the highest inhibitory activity (Figure 5C). In the following experiment, we investigated the anti-inflammatory mechanism of compound **3** in TNF-α/IFN-γ HaCaTs.

### 2.6. Effects of Compounds ***3*** on IL-1β Secretion and the Expression of COX-2/Involucrin/Filaggrin in TNF-α/IFN-γ HaCaTs

**Figure 6:**
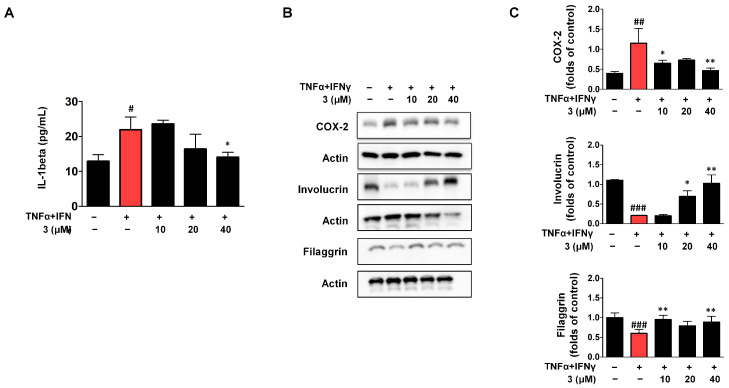
Compound **3** Attenuates TNF-α/IFN-γ-Induced pro-inflammatory Response and Epidermal Barrier Dysfunction. IL-1β secretion (**A**) and the expression of COX-2/involucrin/Filaggrin in TNF-α/IFN-γ HaCaTs (**B**,**C**). The cells were pre-treated with compound **3** for 3 h and then stimulated with TNF-α/IFN-γ for 24 h. The data are represented as the mean ± SD (*n* = 3 independent experiments). ^#^ *p* < 0.05, ^##^ *p* < 0.01, ^###^ *p* < 0.001 vs. untreated control group. * *p* < 0.05, ** *p* < 0.01 vs. TNF-α/IFN-γ-treated group (red column).

A strong pro-inflammatory cytokine, interleukin-1β (IL-1β), plays a pivotal role in the host defense against infection and tissue damage [28]. In our model, IL-1β expression was elevated in TNF-α/IFN-γ-stimulated HaCaT cells, and treatment with compound **3** at 40 μM significantly reduced IL-1β levels (Figure 6A). Cyclooxygenase-2 (COX-2), predominantly expressed in suprabasal keratinocytes, is a key mediator of chronic inflammation [29]. As shown in Figure 6B,C, COX-2 expression was upregulated in response to TNF-α/IFN-γ stimulation. Interestingly, compound **3** reduced COX-2 expression, but this inhibitory effect was evident only at low (10 μM) and high (40 μM) concentrations, with no significant change observed at the intermediate dose (20 μM), suggesting a non-linear or biphasic dose–response pattern. Involucrin, a structural protein expressed in the upper spinous and granular layers, contributes to the formation of the keratinocyte cornified envelope [30,31]. Its expression was downregulated upon TNF-α/IFN-γ stimulation but was partially restored by compound **3** in a dose-dependent manner (Figure 6B,C). Filaggrin, which is essential for maintaining epidermal homeostasis, was also suppressed by TNF-α/IFN-γ stimulation. Similarly to COX-2, filaggrin expression was rescued by compound **3** only at the low and high doses, with the intermediate dose showing no significant effect (Figure 6B,C). This biphasic trend suggests complex regulatory dynamics underlying the dose-dependent effects of compound **3**.

### 2.7. Compound ***3*** Inhibited Activation of the JAK2/STAT (1/3) Signaling Pathway in TNF-α/IFN-γ HaCaTs

**Figure 7:**
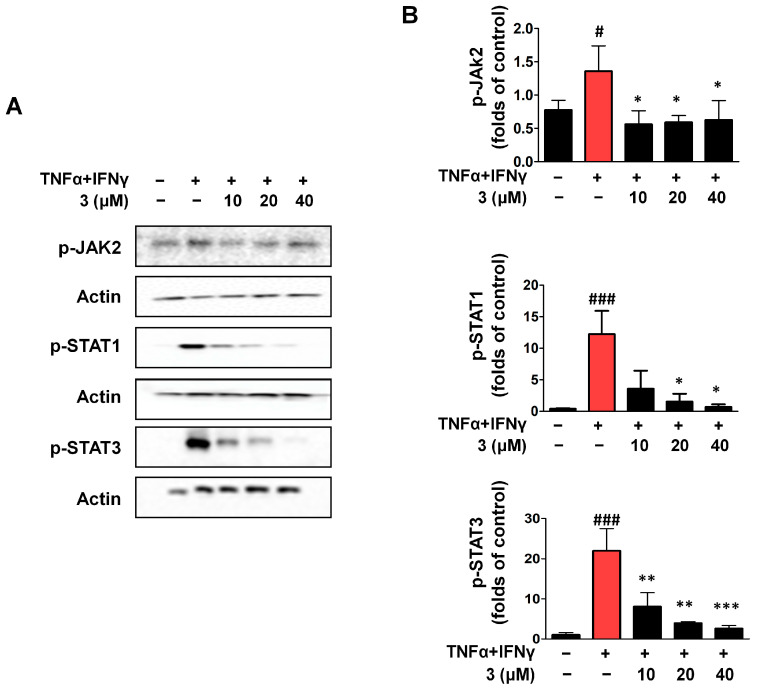
Compound **3** suppresses TNF-α/IFN-γ-induced activation of the JAK/STAT pathway in HaCaT keratinocytes (**A**,**B**). The expressions of p-STAT1, p-STAT3, and p-JAK2 were measured using Western blot. The cells were pre-treated with the compound for 3 h, stimulated with TNF-α/IFN-γ for 15 min, and the cells were collected and lysed. Data are represented as the mean ± SD (*n* = 3 independent experiments). ^#^ *p* < 0.05, ^###^ *p* < 0.001 vs. untreated control group. * *p* < 0.05, ** *p* < 0.01, *** *p* < 0.001 vs. TNF-α/IFN-γ-treated group (red column).

Members of the Janus tyrosine kinase (JAK) family of proteins are essential for the signal transduction initiated by different membrane receptors. Numerous autoimmune and inflammatory illnesses are tightly correlated with persistent activation of the JAK/STAT signaling system [32,33]. Following the activation of cytokine receptors through binding of the respective ligand, JAKs become phosphorylated and promote the phosphorylation and activation of Signal Transducers and Activators of Transcription (STATs). We investigated whether compound **3** had regulatory effects on the JAK/STAT signaling pathway. HaCaTs were stimulated with TNF-α/IFN-γ for 15 min to establish the TNF-α/IFN-γ group. When compared with the TNF-α/IFN-γ group, compound **3** inhibited p-JAK2 and significantly inhibited STAT1 and STAT3 phosphorylation (Figure 7).

### 2.8. Compound ***3*** Inhibited Activation of the NF-κB Signaling Pathway in TNF-α/IFN-γ HaCaTs

**Figure 8:**
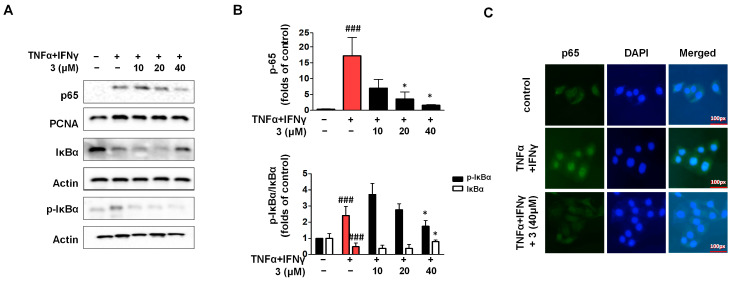
Compound **3** inhibits TNF-α/IFN-γ-induced NF-κB activation in HaCaT keratinocytes (**A**–**C**). The cells were pre-treated with compound **3** for 3 h and then stimulated with TNF-α/IFN-γ for 15 min. The expression of p65, p-IκBα, and IκBα in the fractions were determined using Western blot. The translocation of p65 was detected by immunofluorescence. Data are represented as the mean ± SD (*n* = 3 independent experiments). ^###^ *p* < 0.001 vs. untreated control group. * *p* < 0.05 vs. TNF-α/IFN-γ-treated group (red column).

The nuclear factor kappa-B (NF-κB) pathway is essential for the synthesis of pro-inflammatory proteins, including cytokines, chemokines, and adhesion molecules [34]. The cell solute chelates NF-κB in an inactivated state in response to various stimuli by binding to the inhibitor molecule protein -IκBα. This is followed by phosphorylation and translocation to the nucleus, where further transcription and processing of numerous genes necessary for cellular activity occur [35]. In our experiments, HaCaTs were stimulated with TNF-α/IFN-γ to establish the TNF-α/IFN-γ group, and then we isolated nuclear and cytosolic proteins. When compared with the TNF-α/IFN-γ group. Western blotting results showed that the activity of p65 was inhibited by compound **3** (Figure 8). Phosphorylation and degradation of IκBα were also significantly inhibited by compound **3**. In addition, we detected the nuclear translocation of p65 using IF, which demonstrated the inhibitory effect of compound **3** on p65 translocation into the nucleus.

### 2.9. Compound ***3*** Inhibited the Activation of JNK and ERK in the MAPK Signaling Pathway

**Figure 9:**
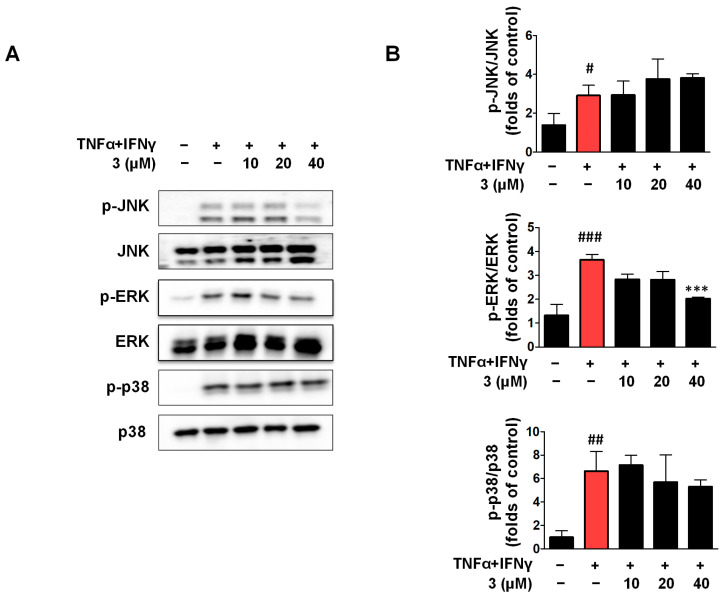
Compound **3** attenuates TNF-α/IFN-γ-induced MAPK pathway activation in HaCaT keratinocytes. The cells were pre-treated with compound **3** for 3 h and then stimulated with TNF-α/IFN-γ (each 5 ng/mL) for 15 min. The expressions of p-JNK, p-ERK, and p-p38 were detected by Western blot (**A**,**B**). Data are represented as the mean ± SD (*n* = 3 independent experiments). ^#^ *p* < 0.05, ^##^ *p* < 0.01, ^###^ *p* < 0.001 vs. untreated control group. *** *p* < 0.001 vs. TNF-α/IFN-γ-treated group (red column).

MAPK is an intracellular enzyme that plays a key role in cellular responses to various stimulants. The mammalian MAPK family includes ERK, p38, and JNK. JNK and p38 are primarily involved in inflammation, apoptosis, and cell growth, whereas ERK is primarily involved in cell growth and differentiation [36]. HaCaTs were stimulated with TNF-α/IFN-γ to set up the TNF-α/IFN-γ group. As shown in Figure 9, when compared with the TNF-α/IFN-γ group, compound **3** only inhibited the phosphorylation of ERK, and Western blot analysis showed that compound **3** affected the phosphorylation of JNK, but this was not statistically significant in contrast to the TNF-α/IFN-γ group. As for p-p38, compound **3** showed no effects when compared with the TNF-α/IFN-γ group.

### 2.10. IL-8 and TARC Secretion Was Inhibited by Compound ***3*** in the 3D-Reconstructed Human Skin Model

**Figure 10:**
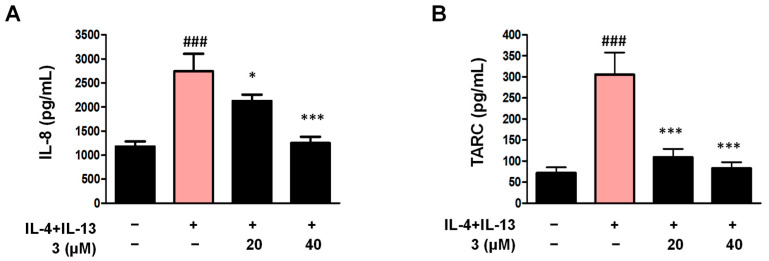
Compound **3** suppresses IL-4/IL-13-induced IL-8 and TARC secretion in a 3D-reconstructed human skin model (**A**,**B**). The 3D-reconstructed human skin model was pre-treated with compound **3** for 2 h, and then stimulated with IL-4/IL-13 (50 ng/mL) for 48 h, and the TARC and IL-8 were detected using an ELISA kit and culture media were from the 3D-reconstructed human skin model. Data are represented as the mean ± SD (*n* = 3 independent experiments). ^###^ *p* < 0.001 vs. untreated control group. * *p* < 0.05, *** *p* < 0.001 vs. TNF-α/IFN-γ-treated group (red column).

We investigated the anti-AD effects of compound **3** by using an interleukin-4 (IL-4)/interleukin-13 (IL-13)-induced 3D-reconstructed human skin model [37]. The skin model KeraSkin™ (BioSolution, Seoul, Republic of Korea) is a commercially available reconstructed human epidermis model prepared from primary normal human keratinocytes [38]. This model consists of a multilayered epidermis with an essential lipid composition similar to that of the human epidermis, expression of skin-specific signals related to epidermal differentiation/proliferation, and cell–cell adhesion [39,40]. After stimulation with IL-4 and IL-13, the 3D-reconstructed human skin model was cultured for 48 h, and the supernatant was used to detect TARC and IL-8. As shown in Figure 10, TARC and IL-8 secretion was significantly increased by IL-4/IL-13 treatment; therefore, compound **3** decreased IL-8 and TARC secretion.

### 2.11. Effects of Compound ***3*** on H&E Staining and Involucrin and Loricrin IHC Analysis in the 3D-Reconstructed Human Skin Model

**Figure 11:**
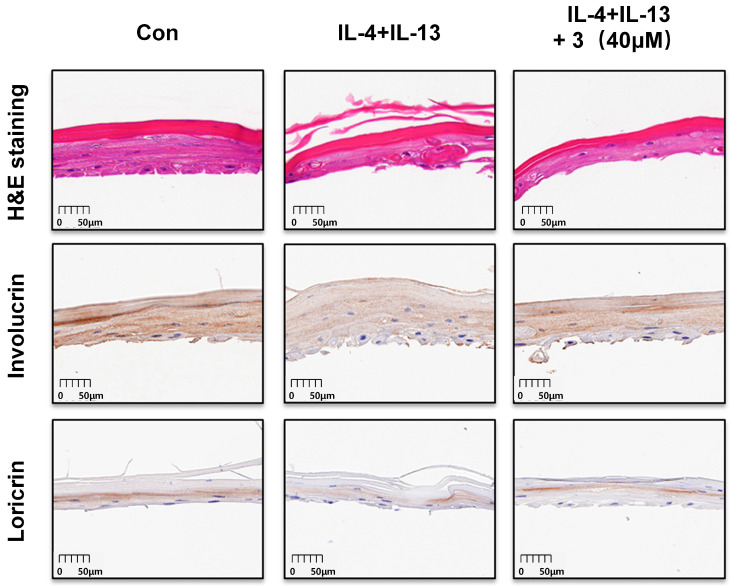
Compound **3** ameliorates epidermal disruption and enhances barrier protein expression in a 3D-reconstructed human skin model. The 3D skin model slices were dyed with H&E and immunohistochemical staining of involucrin and loricrin. was performed (magnification 10.0×). “Con” indicates the untreated control group.

The H&E staining results showed that the 3D-reconstructed skin model had a two-layer structure—the epidermis and partial dermis (Figure 11). IL-4/IL-13 induction caused a disordered arrangement of epidermal cells, significant stratification of the dermis, inflammatory cells in the dermis, and partial changes in cell morphology [39]. Compound **3** partially improved cell morphology and exerted protective effects. The expression of involucrin and loricrin was decreased by IL-4 and IL-13, and IHC analysis showed that compound **3** increased the expression of involucrin and loricrin, indicating a protective effect.

## 3. Discussion

In this study, we demonstrate that compound **3** (desrhamnosyl acteoside), isolated from *Digitalis purpurea* L., exhibits significant anti-inflammatory and barrier-restorative effects in TNF-α/IFN-γ-stimulated HaCaT keratinocytes and a 3D-reconstructed human skin model. Specifically, compound **3** suppressed the expression of pro-inflammatory cytokines (e.g., IL-6 and IL-8), chemokines (e.g., TARC and RANTES), and inflammatory proteins such as COX-2. Moreover, it modulated key signaling pathways, including NF-κB, JAK2/STAT1, and MAPKs. Notably, compound **3** also restored the expression of epidermal barrier proteins such as filaggrin and involucrin, which were reduced by inflammatory stimulation. These findings suggest that compound **3** may exert dual effects, including anti-inflammatory and barrier-repair effects on skin, offering potential therapeutic relevance for inflammatory skin disorders such as atopic dermatitis.

Natural products, recognized for their ability to modulate multiple signal transduction pathways rather than acting through a single mechanism, exhibit substantial potential pharmacological effects, a multitarget regulatory strategy that has been widely acknowledged [41]. Compared to synthetic drugs, their diverse chemical scaffolds and broad biological origins make them appealing and cost-effective sources of novel therapeutics. *Digitalis purpurea* L., which is widely distributed and accessible, presents a valuable source for isolating bioactive compounds. While prior studies have extensively reported its cardiotonic glycosides [42,43,44], its potential application in skin disease treatment remains largely unexplored.

Our study aims to expand the currently limited understanding of the pharmacological effects of phenylethanoid glycosides derived from *Digitalis purpurea* L. on skin inflammation. Among the eight isolated compounds, desrhamnosyl acteoside was identified as the most potent in suppressing keratinocyte-mediated inflammation, both in monolayer cultures and a 3D-reconstructed human skin model. Desrhamnosyl acteoside is a hydroxytyrosol-based glycoside, structurally derived from acteoside (verbascoside) through the loss of a rhamnose moiety, resulting in the retention of hydroxytyrosol conjugated with caffeic acid and glucose. Although hydroxytyrosol is a well-established antioxidant found in olive oil, known for its anti-inflammatory and skin-protective properties [45,46], the biological activity of desrhamnosyl acteoside itself has not been previously investigated in the context of skin diseases. In this study, we demonstrate for the first time that desrhamnosyl acteoside significantly attenuates inflammatory responses in keratinocytes by modulating NF-κB, JAK2/STAT1, and MAPK signaling pathways and selectively regulating ERK phosphorylation. These findings highlight desrhamnosyl acteoside as a novel bioactive compound with potential therapeutic relevance for inflammatory skin disorders, distinct from the effects attributed solely to hydroxytyrosol.

One advantage of our experimental approach is the use of a three-dimensional (3D) reconstructed human skin model, which provides a more physiologically relevant environment than traditional two-dimensional (2D) cell cultures. This model enables a more representative evaluation of compound effects on skin tissue architecture and function and has been increasingly employed in dermatological research [36,37,38,39,40]. However, a limitation of this model is that it does not fully replicate the complexity of in vivo human skin, particularly with respect to immune system interactions and the presence of additional cell types such as dermal fibroblasts, melanocytes, and immune cells.

Our finding that desrhamnosyl acteoside from *D. purpurea* L. exhibits anti-inflammatory and protective effects is novel in the context of skin disease research. While previous studies have focused on the cardiotonic effects of *D. purpurea* L. compounds, our study reveals a new potential application for this compound as a candidate adjuvant therapy for atopic dermatitis (AD) or drugs related to skin inflammation. This discovery expands the scope of potential therapeutic applications of *D. purpurea* L. and aligns with the growing interest in natural products as sources of novel therapeutics for skin diseases [47]. Overall, our study contributes to the understanding of the pharmacological potential of *D. purpurea* and opens new avenues for future research in the field of skin disease treatment.

In the pathology of atopic dermatitis (AD), keratinocyte-secreted inflammatory mediators, such as inflammatory factors, chemokines, and functional proteins, are pivotal, as extensively documented in the previous literature. Type 2 immune cytokines, notably IL-4 and IL-13, have been shown to be central in chemokine production, skin barrier disruption, antimicrobial peptide (AMP) inhibition, and allergic inflammation processes. Through STAT6 activation, IL-4 and IL-13 decrease the expression of key structural proteins like filaggrin, loricrin, and involucrin, and also impact the lipid content essential for skin barrier function, leading to increased trans-epidermal water loss (TEWL) that is a common marker for predicting AD [48,49]. Additionally, their stimulation of peripheral pruritus sensory neurons results in itchy skin, a distressing hallmark of AD.

Our present study, which investigates the anti-inflammatory and cytoprotective effects of eight compounds isolated from *Digitalis purpurea* L. on HaCaT cells, has both strengths and limitations. A significant advantage is that using HaCaT cells provides a standardized in vitro model to study the effects of the compounds, allowing for precise control of experimental conditions and the ability to isolate the effects of the compounds on keratinocytes. This approach is consistent with many previous studies that have utilized HaCaT cells to study skin inflammation and related mechanisms [50]. However, a limitation is that HaCaT cells are a transformed cell line and may not fully represent the behavior of primary keratinocytes in vivo, potentially affecting the translation of our findings to the clinical setting.

The key result of our study is that compound **3**, desrhamnosyl acteoside, exhibited an anti-inflammatory effect on the skin by inhibiting the release of inflammatory factors and chemokines, downregulating inflammatory proteins, and regulating the NF-κB, JAK2/STAT1, and MAPK signaling pathways, is both novel and related to existing literature. While previous research has focused on the roles of cytokines and signaling pathways in AD pathology, our study identifies a natural compound from *Digitalis purpurea* L. that can modulate these pathways. This finding expands on the understanding of potential therapeutic targets for AD and aligns with the growing interest in natural products as sources of anti-inflammatory agents for skin diseases. It provides a new lead for the development of AD therapies but also emphasizes the need for further research, such as in vivo studies, to confirm the efficacy and safety of desrhamnosyl acteoside in treating AD.

Our experimental results are closely related to the existing literature. The observation that desrhamnosyl acteoside inhibited the phosphorylation of JAK2, STAT1, and STAT3 indicates its potential anti-inflammatory effect mediated by regulating the JAK2/STAT1 signaling pathway, which is consistent with the known role of JAK inhibitors in AD treatment. Regarding the NF-κB pathway, our finding that desrhamnosyl acteoside significantly inhibited the NF-κB signaling is in line with the understanding that NF-κB is a crucial regulator of immune and inflammatory responses in AD. For the MAPK signaling pathway, based on previous research where the ERK pathway is involved in epidermal barrier function and the JNK and p38 pathways are associated with immune diseases and cancer [51], our discovery that desrhamnosyl acteoside selectively inhibited the phosphorylation of ERK while having no significant impact on p-p38 and p-JNK is novel. This suggests that desrhamnosyl acteoside may influence epidermal barrier function through selective modulation of ERK phosphorylation, providing a new perspective on the therapeutic potential of this compound for AD.

The 3D-reconstructed human skin model used in this study is KeraSkin™, which is derived from primary human keratinocytes cultured at the air–liquid interface. This model faithfully replicates the structural stratification of native human skin, from the basal layer to the cornified layer, and incorporates essential barrier components such as filaggrin and ceramides [52]. It has been validated by the OECD Test Guideline 439 as an alternative to animal testing for evaluating skin irritation and inflammation [53]. KeraSkin™ enables the modeling of cytokine-induced inflammation (e.g., IL-4/IL-13), biomarker quantification (e.g., IL-8 and TARC), and histological analysis and is suitable for high-throughput screening under standardized protocols. Using this model, we examined the anti-inflammatory effects of desrhamnosyl acteoside isolated from *Digitalis purpurea* L. The results demonstrated a significant reduction in inflammatory markers, indicating its potential therapeutic value. However, our findings are limited by the need for additional experiments to elucidate the mechanisms of the 3D-reconstructed human skin models. Nonetheless, our results provide a significant foundation suggesting that desrhamnosyl acteoside may exhibit substantial anti-inflammatory effects on the skin in both in vivo experiments and clinical trials.

## 4. Materials and Methods

### 4.1. Sample Preparation

Eight compounds were extracted from *D. purpurea* L., separated, purified, and stored at the Natural Product Laboratory of Chosun University. The separation and structural identification of the compounds are described in our previous papers [22,54,55].

### 4.2. Cell Culture

Human epidermal keratinocytes (HaCaTs) were purchased from AddexBio (San Di-ego, CA, USA) and cultured in Dulbecco’s modified eagle medium (DMEM) with 10% fetal bovine serum (FBS; Gibco, MA, USA,). Human recombinant TNF-α, IFN-γ, and pre-coated enzyme-linked immunosorbent assay (ELISA) kits for IL-8, IL-6, macrophage derived chemokine (MDC), and RANTES were purchased from BioLegend (San Diego, CA, USA). Antibodies against intercellular adhesion molecule (ICAM-1), Involucrin, COX-2, Loricrin, p-IκBα, IκBα, p65, p-JAK2, p-STAT1, p-STAT3, p-ERK, ERK, p-JNK, JNK, actin, PCNA, HRP-conjugated anti-mouse, and anti-rabbit IgG were purchased from Santa Cruz Biotechnology (Santa Cruz, CA, USA) and Cell Signaling Technology (Danvers, MA, USA).

### 4.3. 2,5-Iphenyl-2H-Tetrazolium Bromide (MTT) Assay

HaCaTs were incubated with eight compounds isolated from *D. purpurea* L. (10–40 μM). After 24 h, the cells were treated with 0.5 mg/mL MTT for 1 h, and the formazan formed was dissolved in dimethyl sulfoxide (DMSO). The absorbance of dissolved formazan was measured at 540 nm using an ELISA microplate reader (Molecular Devices, San Jose, CA, USA).

### 4.4. IL-6, IL-8, MDC, and RANTES Detection in Cell Culture Supernatants

HaCaTs were seeded in 24-well plates, pre-treated for 3 h with various compounds from *D. purpurea* L., and then stimulated with TNF-α and IFN-γ (5 ng/mL each) for an additional 24 h. The manufacturer’s instructions were followed to quantify IL-6, IL-8, RANTES, and MDC levels in the cell supernatants using an ELISA kit.

### 4.5. Protein Extracts

HaCaTs were pre-treated with compound **3** for 3 h at 10–40 μM. The cells were stimulated with TNF-α/IFN-γ (5 ng/mL each) for 24 h to perform a Western blot analysis to analyze ICAM-1, COX-2, and involucrin. The cells were stimulated with TNF-α/IFN-γ for 15 min to determine the p-IκBα, IκBα, p65, p-JAK2, p-STAT1/p-STAT3, p-p38, and p-JNK levels. The cells were then removed and lysed using radioimmunoprecipitation assay (RIPA) buffer for total protein analysis. Cells were harvested, and nuclear and cytoplasmic proteins were extracted using a Nuclear Extraction Kit (Cayman Chemical, Ann Arbor, MI, USA) in accordance with the manufacturer’s recommendations. Proteins were stored at −80 °C before use.

### 4.6. Western Blot Analysis

Protein samples were separated by sodium dodecyl sulfate-polyacrylamide gel electrophoresis (SDS-PAGE) and transferred to nitrocellulose membranes. The membrane was blocked with 5% skim milk for 60 min, incubated with the appropriate primary antibodies at a dilution of 1:1000 overnight at 4 °C, and then incubated at room temperature (25 °C) for 1 h with a dilution of 1:5000 with a secondary antibody conjugated to HRP. Specific proteins were detected using an enhanced chemiluminescence solution after washing with TBST. ImageJ software (ImageJ 1.54, National Institutes of Health, Rockville, MD, USA) was used to analyze the optical density of the bands. For each band, the integrated density was measured after converting the image to 8-bit and inverting it. Background intensity was subtracted by selecting an area near each band. All target protein bands were normalized to the corresponding β-actin, PCNA, or house-keeping protein bands. Quantified values from three independent experiments (n = 3) were expressed as mean ± standard deviation, and statistical significance was determined using one-way ANOVA with appropriate post hoc tests. No membrane was cut before hybridization with primary and secondary antibodies.

### 4.7. Immunofluorescence (IF)

IF was used to identify the translocation of NF-κB. HaCaT cells were plated on glass chamber slides, pre-treated with compound **3** (40 μM) for 3 h, and stimulated with TNF-α/IFN-γ for 15 min. Cells were fixed in 4% paraformaldehyde, transparented, blocked, and then incubated with NF-κB antibodies and secondary antibodies that were fluorescein (FITC)-labeled. After 5 min of incubation, the nuclei were stained with 4’,6-diamidino-2-phenylindole (DAPI) and mounted on coverslips on glass slides. Images were captured using a fluorescence microscope (Nikon Optical, Tokyo, Japan).

### 4.8. Three-Dimensional (3D)-Reconstructed Human Skin Model

The 3D-reconstructed human skin model (KeraSkin™) was purchased from BioSolution (Seoul, Republic of Korea). It is composed of primary human keratinocytes cultured at the air–liquid interface to mimic native epidermal stratification and barrier function. The model is OECD TG 439-validated and widely employed in dermatological research for evaluating inflammation, barrier restoration, and drug efficacy under animal-free conditions [52,53,54]. The tissue was placed in a six-well plate prefilled with 0.9 mL of medium for 24 h. Next, the tissue was pre-treated with compound **3** (20 and 40 μM) for 3 h and then treated with IL-4/IL-13 (per 50 ng/mL) for 48 h. The culture medium was collected and stored at −80 °C, and the skin tissues were preserved in 4% paraformaldehyde.

### 4.9. Hematoxylin and Eosin (H&E) Staining and Immunohistochemical (IHC) Analysis

The paraffin fixed 3D-reconstructed human skin tissue was cut into sections with 5-μm thickness. H&E staining was performed. Skin sections were stained with involucrin and loricrin antibodies and biotinylated rabbit anti-goat IgG and images of the cells were captured (Nikon Optical, Tokyo, Japan).

### 4.10. Statistical Analysis

Data are presented as mean ± standard deviation (SD). One-way analysis of variance (ANOVA) followed by Tukey’s post hoc test was performed using GraphPad Prism software (version 8.0). Statistical significance was set at * *p* < 0.05, ** *p* < 0.01, and *** *p* < 0.001. Each experiment was repeated thrice.

## 5. Conclusions

Our study investigates the anti-inflammatory effect on the skin of compounds isolated from *D. purpurea* L. in TNF-α/IFN-γ-induced HaCaTs and a 3D-reconstructed human skin model. Desrhamnosyl acteoside exhibited an anti-inflammatory effect on the skin, which inhibited the release of inflammatory factors and chemokines, inhibited the expression of inflammatory proteins, and regulated NF-κB, JAK2/STAT1, and MAPK signaling pathways in HaCaTs. It also exhibits skin-protective activity in an IL-4/IL-13-induced 3D-reconstructed human skin model. These results demonstrate the potential of desrhamnosyl acteoside isolated from *D. purpurea* L. to inhibit skin inflammatory diseases and its promising prospects as a future treatment agent.

## Data Availability

The data presented in this study are available on request from the corresponding author.

## Abbreviations

Following abbreviations are used in this manuscript:

AD	Atopic dermatitis
*Digitalis purpurea* L.	*D. Purpurea* L.
3D	Three-dimensionally
2D	Two-dimensionally
TNF-α	Tumor necrosis factor
IFN-γ	interferon-γ
COX-2	Cyclooxygenase-2
NF-κB	Nuclear factor kappa-B
JAK	Janus kinase
IκBα	I kappa B-alpha
STAT	Signal Transduction and Transcription Activating Factor
MAPK	Mitogen-activated protein kinase
TJ	Tight junctions
AMPs	Antimicrobial peptides
RANTES	Regulated upon Activation Normal T-cell Expressed and Secreted
TARC	Thymus to release activated regulatory chemokine
MDC	Macrophage-derived chemokine
IL-1β	Interleukin-1β
IL-6	Interleukin-6
IL-8	Interleukin-8
IL-4	Interleukin-4
IL-13	Interleukin-13
TEWL	Trans-epidermal water loss
ICAM-1	Intercellular adhesion molecule-1
